# Microbiome Dysbiosis Shows Strong Association of Gut-Derived Altered Metabolomic Profile in Gulf War Chronic Multisymptom Illness Symptom Persistence Following Western Diet Feeding and Development of Obesity

**DOI:** 10.3390/ijms24044245

**Published:** 2023-02-20

**Authors:** Dipro Bose, Vitalli Stebliankin, Trevor Cickovski, Punnag Saha, Ayushi Trivedi, Subhajit Roy, Madhura More, Ashok Tuteja, Kalai Mathee, Giri Narasimhan, Saurabh Chatterjee

**Affiliations:** 1 Environmental Health and Disease Laboratory, Department of Environmental and Occupational Health, Program in Public Health, Susan and Henry Samueli College of Health Sciences, University of California, Irvine, CA 92697, USA; 2 Knight Foundation School of Computing and Information Sciences, College of Engineering and Computing, Florida International University, Miami, FL 33199, USA; 3 Division of Internal Medicine, University of Utah School of Medicine, Salt Lake City VAMC, Salt Lake City, UT 84132, USA; 4 Department of Human and Molecular Genetics, Herbert Wertheim College of Medicine, Florida International University, Miami, FL 33199, USA; 5 Biomolecular Sciences Institute, Florida International University, Miami, FL 33199, USA; 6 Department of Medicine, Infectious Disease, UCI School of Medicine, Irvine, CA 92697, USA; 7 VA Research and Development, VA Long Beach Health Care System, Long Beach, CA 90822, USA

**Keywords:** microbiome, metabolome, gut, chronic multisymptom illness, whole-genome sequencing

## Abstract

The pathophysiology of Gulf War Illness (GWI) remains elusive even after three decades. The persistence of multiple complex symptoms along with metabolic disorders such as obesity worsens the health of present Gulf War (GW) Veterans often by the interactions of the host gut microbiome and inflammatory mediators. In this study, we hypothesized that the administration of a Western diet might alter the host metabolomic profile, which is likely associated with the altered bacterial species. Using a five-month symptom persistence GWI model in mice and whole-genome sequencing, we characterized the species-level dysbiosis and global metabolomics, along with heterogenous co-occurrence network analysis, to study the bacteriome–metabolomic association. Microbial analysis at the species level showed a significant alteration of beneficial bacterial species. The beta diversity of the global metabolomic profile showed distinct clustering due to the Western diet, along with the alteration of metabolites associated with lipid, amino acid, nucleotide, vitamin, and xenobiotic metabolism pathways. Network analysis showed novel associations of gut bacterial species with metabolites and biochemical pathways that could be used as biomarkers or therapeutic targets to ameliorate symptom persistence in GW Veterans.

## 1. Introduction

To date, GW Veterans continue to be afflicted with metabolic disorders such as diabetes and obesity, along with the symptom persistence of GWI. A total of 29.6% of GW Veterans suffer from obesity [1]. Due to a sedentary lifestyle and dietary patterns that mainly comprise the Western-style diet, there are increased risks of developing arthritis, cardiovascular disease, and metabolic disease among GW Veterans [2]. With the continued challenge of identifying a proper treatment regime to alleviate the symptom persistence associated with GWI, it is important to utilize developing non-invasive multi-omics techniques for designing therapeutic strategies.

In our previous studies, we have established the role of altered gut bacteriome playing a significant role in the pathology of GWI [3,4,5,6,7]. The gut bacterial community can be influenced by a large number of factors including diet, age, and lifestyle [8]. In our recent studies, we have reported that a Western diet exacerbates GW chemical-induced gastrointestinal and hepatic inflammation, increasing neurodegeneration in an established GWI murine persistence model [9]. Studies have also reported that these factors impact the metabolic activity of the resident gut bacteria, thereby affecting its colonization and survival [10]. Thus, there is a growing trend of using a multi-omics approach and identifying methods to associate the altered gut bacterial population with altered metabolites. The altered metabolites have direct implications for the host’s health and can form early biomarkers for diseases such as inflammatory bowel disease, nonalcoholic fatty liver disease, and neurological diseases [11,12,13]. Researchers have developed mathematical algorithms for identifying the correlation between the host’s metabolomic profile with that of the gut bacterial composition.

Metabolomic studies were performed in GWI murine models, where the researchers reported major alteration of the plasma lipid metabolism with increased accumulation of sphingomyelin, phosphatidylcholine, and decreased fatty acid-binding protein 3. These changes persisted for 150 days after the initial exposure to representative GW chemicals permethrin (Per) and pyridostigmine bromide (PB) [14]. However, to date, there are no studies that report global metabolomic analysis as compared to the existing lipidomic analysis or an association study between gut bacteria and metabolites in GWI.

In this study, using whole-genome sequencing and global metabolomic analysis along with heterogenous co-occurrence network analysis, we investigated the effect of a Western diet in altering the metabolomic profile of the host in an established GWI murine 20-week persistence model that closely represents the health condition of current GW Veterans. We also hypothesized that statistically significant associations would be identified in the mice groups exposed to representative GW chemicals, as well as in those that were fed with a Western diet and were pre-exposed to GW chemicals.

## 2. Results

### 2.1. Western Diet Significantly Altered Gut Bacteriome Composition in Mice Exposed to GW Chemicals

**Figure 1:**
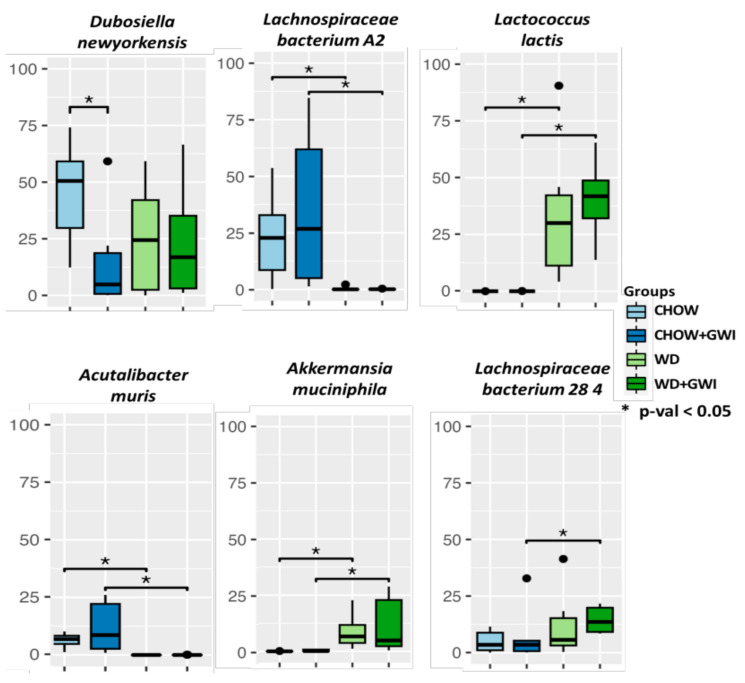
Western diet-induced obesity exacerbates gut dysbiosis in underlying GWI conditions. Box plots showing the relative abundance of significantly altered bacteria at the species level in Chow, Chow + GWI, WD, and WD + GWI groups. *p*-values were calculated by the Mann–Whitney test, where *p* < 0.05 was considered statistically significant. The black dots are used to denote the outlier data points.

We have reported in our previous study that groups of mice that were administered with representative GW chemicals PB and Per and developed obesity induced by a Western diet showed observable changes in the gut bacterial species diversity and abundance [9]. The alpha diversity measured by the Shannon index showed a significant change between the Chow (mice group fed with chow diet) and WD (mice group fed with Western diet) groups (*p* ≤ 0.001). However, the change between Chow + GWI (mouse group exposed to GW chemicals PB and Per and fed with chow diet) and WD + GWI (mouse group exposed to GW chemicals PB and Per and fed with Western diet) groups was non-significant. Our results of Bray–Curtis beta-diversity analysis showed Chow and Chow + GWI groups formed an independent cluster from the WD and WD + GWI groups [9]. At the species-level analysis, we found that relative abundances of *Lactococcus lactis, Lachnospiraceae bacterium 28-4,* and *Akkermansia muciniphila* were significantly increased in the WD + GWI group compared to Chow + GWI (*p* < 0.05) (Figure 1). Relative abundances of *Lachnospiraceae bacterium A2* and *Acutalibacter muris* were significantly decreased in the WD + GWI group compared to the Chow + GWI group (*p* < 0.05). However, the relative abundance of *Dubosiella newyorkensis* was decreased in the Chow + GWI group compared to the Chow group (*p* < 0.05) and showed no significant changes in the Western diet-exposed groups.

### 2.2. Fecal Metabolomic Analysis Showed Significantly Altered Metabolite Profile in Mice Exposed to GW Chemicals and Western Diet

We performed a global metabolomic analysis from Metabolon Inc. (Morrisville, NC, USA) using fecal pellets collected from all the experimental mice to identify the major altered biochemical pathways and the related metabolites. There were 1025 metabolites identified, among which 859 compounds were known and 166 compounds were unknown.

**Figure 2:**
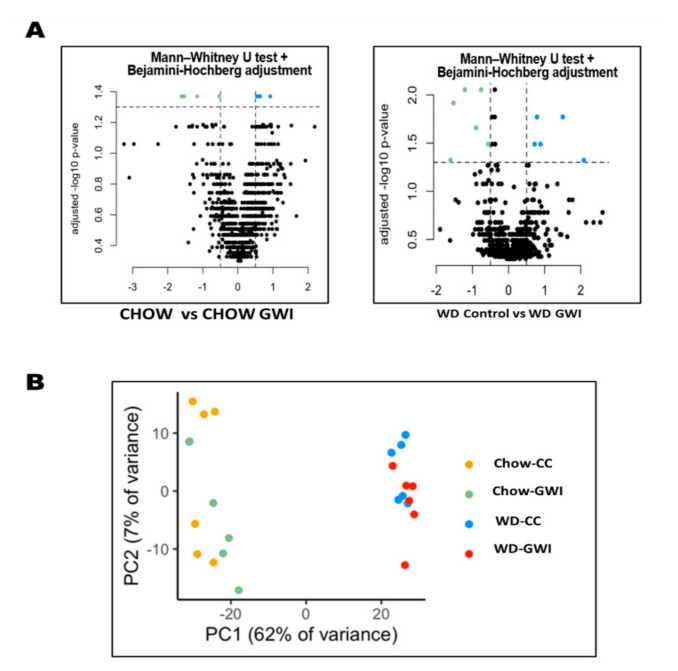
Western diet-induced obesity alters metabolomic profile in underlying GWI conditions. (**A**) Volcano plot showing the distribution of metabolites in the Chow, Chow + GWI, WD l, and WD + GWI groups; (**B**) PCA plot showing the β-diversity of analyzed fecal metabolites in Chow, Chow + GWI, WD and WD + GWI groups.

The volcano plot shows that Western diet-exposed groups had more significant fold changes in the metabolites compared to the chow diet-exposed mice groups (Figure 2A). PCA analysis was performed to study the beta diversity of the metabolites among the four mice groups. The PCA analysis showed the Chow and Chow + GWI groups and WD and WD + GWI groups overlap. However, the overall chow diet mice groups and the Western diet mice groups formed an independent cluster (Figure 2B). The pattern was similar to the gut bacterial beta diversity [9].

**Figure 3:**
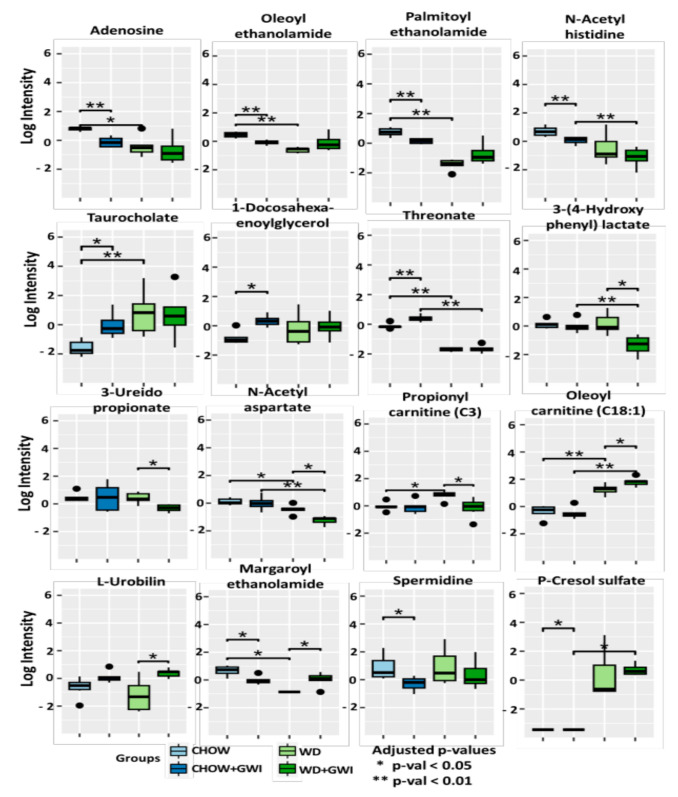
Significantly altered metabolites across the experimental groups obtained from the global metabolomic analysis. Box plots showing the significantly altered metabolites in the Chow, Chow + GWI, WD, and WD + GWI groups. The box plots were constructed using log-transformed raw metabolite concentrations (based on ion counts). *p*-values were calculated by the Mann-Whitney test, where *p* < 0.05 was considered statistically significant. The black dots are used to denote the outlier data points.

Further investigation of the effect of Western diet exposure in underlying GW conditions identified 16 metabolites that were significantly altered among the experimental groups (Figure 3). The metabolites majorly affected the lipid, amino acid, nucleotide, vitamin, and xenobiotic metabolism pathways.

In the lipid metabolism pathways, we found that oleoyl ethanolamide, palmitoyl ethanolamide, and margaroyl ethanolamide were metabolites that were involved with endocannabinoid metabolism [15,16]. The fold change of the three metabolites was significantly lowered in Chow + GWI groups compared to Chow (*p* < 0.05). However, the fold change of margaroyl ethanolamide was significantly increased in the WD + GWI group compared to the WD group (*p* < 0.05). The fold change of taurocholate (involved in primary bile acid metabolism) and 1-docosahexaenoyl glycerol (which is involved in monoacyl glycerol metabolism) was found to be significantly increased in the Chow + GWI group compared to the Chow group (*p* < 0.05). Propionyl carnitine (C3) and oleoyl carnitine (C18:1), which are involved in fatty acid metabolism, were also significantly increased in the WD group compared to the Chow group (*p* < 0.05). Among the metabolites involved in amino acid pathways, the fold change of N-acetyl histidine (involved in histidine metabolism), 3-(4-hydroxyphenyl) lactate (involved in tyrosine metabolism), and N-acetyl aspartate (involved in aspartate metabolism) were significantly decreased in the WD + GWI group compared to the Chow + GWI group (*p* < 0.05). In addition, the fold change of spermidine, which is a metabolite involved in polyamine metabolism, was significantly decreased in the Chow + GWI group compared to the Chow group (*p* < 0.05). There were alterations in two metabolites, adenosine and 3-ureidopropionate, belonging to the nucleotide metabolic pathway. Adenosine, which is involved in purine metabolism, was significantly decreased in the Chow + GWI group compared to the Chow group (*p* < 0.05) [17]. The metabolite 3-ureidopropionate, which is involved in the uracil-containing pyrimidine metabolism, was significantly decreased in the WD + GWI group compared to the WD group (*p* < 0.05). Threonate, which is involved in the ascorbate and aldarate metabolism, was significantly decreased in the WD + GWI group compared to the Chow + GWI group (*p* < 0.05) [18]. A metabolite involved with xenobiotic metabolism, p-cresol sulfate, was significantly increased in WD + GWI groups compared to the Chow + GWI group (*p* < 0.05). Finally, L-urobilin, which is involved in hemoglobin metabolism, was found to be significantly increased in the WD + GWI group compared to the WD group.

### 2.3. Association Study Showed That Altered Metabolites Were Correlated with Gut Bacterial Species

We investigated whether the altered gut bacterial species were associated with altered metabolites and biochemical pathways. Heterogenous network and pathway analysis showed that in each experimental group, certain bacterial species were positively correlated with metabolites (Spearman correlation, where *p* ≤ 0.05 was considered significant).

**Figure 4:**
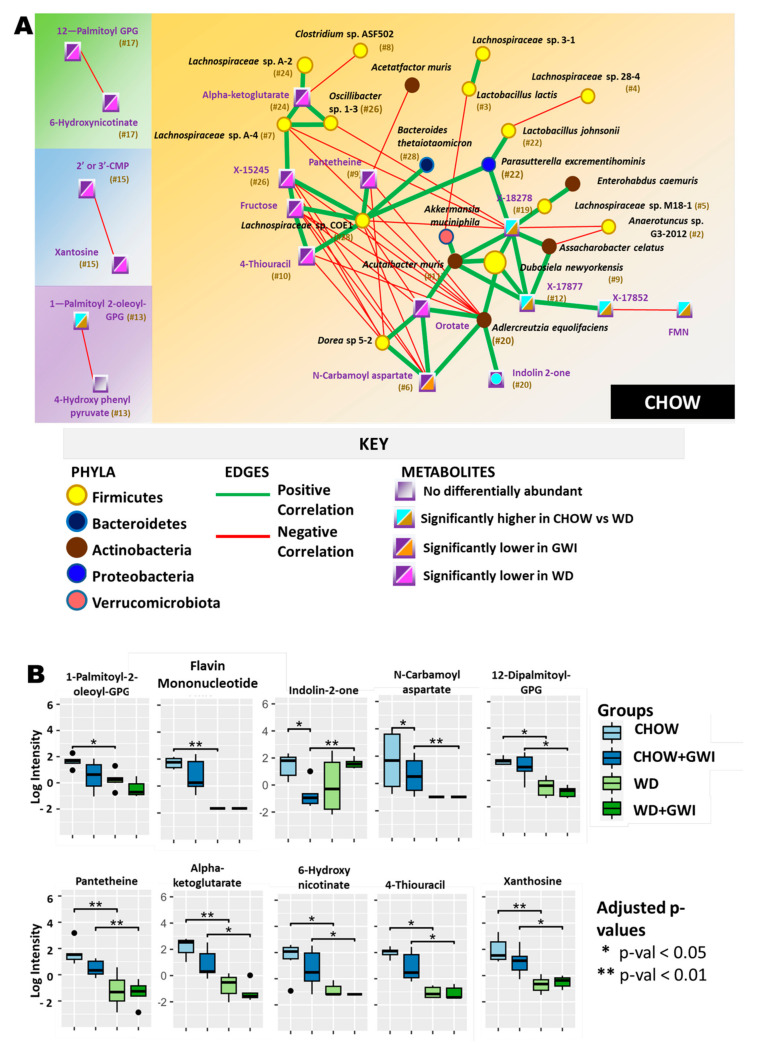
Heterogenous network showing an association between altered gut bacteria and metabolites in the Chow group. (**A**) The figure shows the heterogeneous co-occurrence networks for the Chow Control group. Circular nodes represent microbes in these networks, and squares represent metabolites. Microbe nodes (circles) have been colored by phylum (yellow = Firmicutes, brown = Actinobacteria, blue = Proteobacteria, violet = Bacteroidetes), with size proportional to their abundance. Metabolite nodes (squares) have been colored based on the sample set(s) where they are differentially abundant; otherwise, they are grey. Green edges represent positive correlations, and red edges represent negative correlations. The Fruchterman–Reingold algorithm has been used for visualization, keeping positively correlated entities in close proximity. Nodes have been labeled with their microbe or metabolite name, with a ranked centrality (importance) computed using Ablatio Triadum, which has been shown to uncover important driver, villain, and bridge nodes in signed and weighted biological networks. (**B**) Box plot showing network-specific metabolites that were altered. The box plots were constructed using log-transformed raw metabolite concentrations (based on ion counts). *p*-values were calculated by the Mann–Whitney test, where *p* < 0.05 was considered statistically significant. The black dots are used to denote the outlier data points.

Chow group appeared as an “overarching” control network (Figure 4A). A bacterial species that was distinct in this network was *Adlercreutzia equolifaciens* (ranked #20 by Atria), and it was found to be positively correlated with three metabolites that also appeared in the Kyoto Encyclopedia of Genes and Genomes (KEGG). Two of these metabolites were indolin-2-one (ranked #20) and N-carbamoylaspartate (ranked #5). Orotate, a metabolite associated with pyrimidine metabolism and biosynthesis of cofactors, appeared twice [19]. It is interesting to note that N-carbamoylaspartate was present in the Chow but not the Chow + GWI group, and N- carbamoylaspartate is also associated with the alanine, aspartate, and glutamate metabolism pathways which are again associated with *Adlercreutzia equolifaciens*. Research on N-carbamoylaspartate is limited, but it is involved in the biosynthesis of glutamate, which is one of the essential amino acids and among the most abundant neurotransmitters, as a crossroad of multiple metabolite pathways [20]. Alpha-ketoglutarate (ranked #24), which is reported as a key molecule for gut metabolism [21], was also found in the Chow group network only. There were 10 Chow group network-specific metabolites identified, where the fold change of these metabolites was highest in the Chow group (Figure 4B).

**Figure 5:**
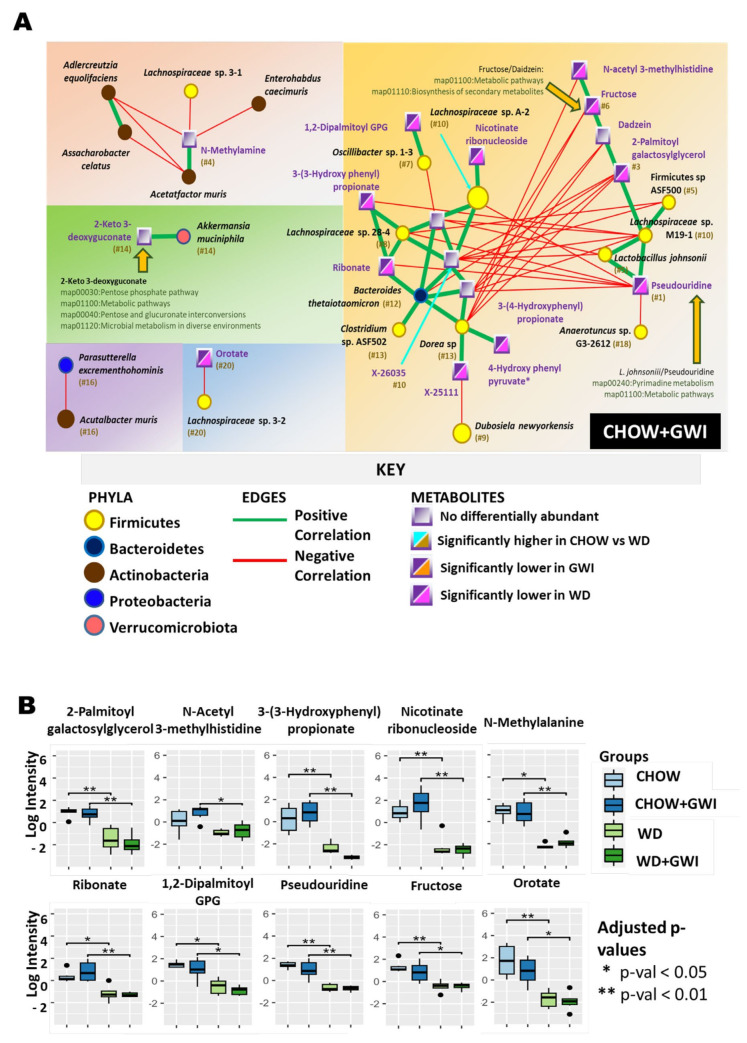
Heterogenous network showing an association between altered gut bacteria and metabolites in the Chow + GWI group. (**A**) The figure shows the heterogeneous co-occurrence networks for the Chow GWI group. Circular nodes represent microbes in these networks, and squares represent metabolites. Microbe nodes (circles) have been colored by phylum (yellow = Firmicutes, brown = Actinobacteria, blue = Proteobacteria, violet = Bacteroidetes), with size proportional to their abundance. Metabolite nodes (squares) have been colored based on the sample set(s) where they are differentially abundant; otherwise, they are grey. Green edges represent positive correlations, and red edges represent negative correlations. The Fruchterman–Reingold algorithm has been used for visualization, keeping positively correlated entities in close proximity. Nodes have been labeled with their microbe or metabolite name, with a ranked centrality (importance) computed using Ablatio Triadum, which has been shown to uncover important driver, villain, and bridge nodes in signed and weighted biological networks. Amber arrows point to any positive correlations that are also backed up by documented pathways in the database KEGG. (**B**) Box plot showing network-specific metabolites that were altered. The box plots were constructed using log-transformed raw metabolite concentrations (based on ion counts). *p*-values were calculated by the Mann–Whitney test, where *p* < 0.05 was considered statistically significant. The black dots are used to denote the outlier data points.

In the Chow + GWI group network, we observed the appearance of *Akkermansia muciniphila*, which was positively correlated with 2-keto-3-deoxygluconate (ranked #14), a metabolite known to be involved in pentose and glucuronate pathways (Human Metabolome Database No. 0001353) (Figure 5A). Glucuronate is important for plant and animal metabolism. One of the associated pathways, i.e., the pentose phosphate pathway, is fundamental to cellular metabolism [22]. Additionally, daidzein, associated with fructose metabolism pathways, and known for its protective role in intestinal health was observed in this network [23]. *Lactobacillus johnsonii*, a known probiotic bacterium [24], appeared as a central bacterium and was connected with the top metabolite pseudouridine (ranked #1) through a supported KEGG pathway involving pyrimidine metabolism. We observed that the acute toxin N-methylamine (ranked #4) was negatively correlated with five microbes, four of which belonged to the *Actinobacteria* phylum. This included *Adlercreutzi equolifaciens*, which was found to play a much more central role in the Chow group network and had positive correlations to many other microbes and metabolites. The network analysis suggests that any potential effect of GWI pathology on N-methylamine concentrations and subsequent impacts on the role of *Adlercreutzia equolifaciens* are worthy of further exploration. There were 10 network-specific metabolites identified in Chow + GWI group(Figure 5B).

**Figure 6:**
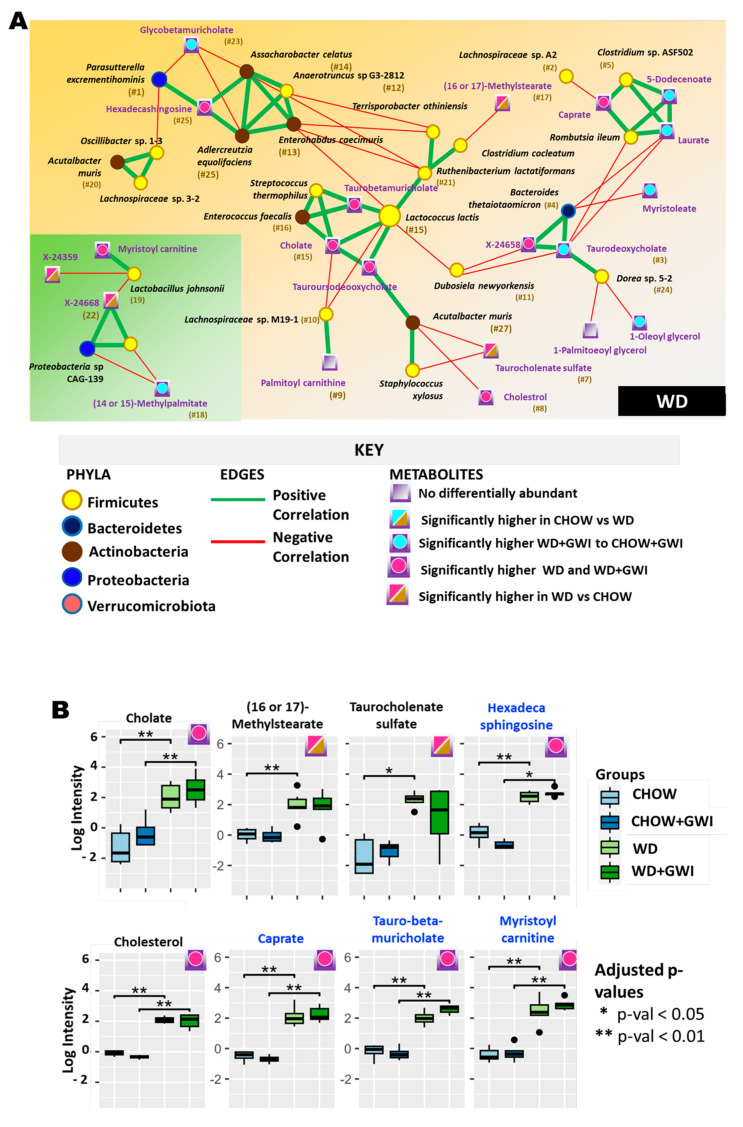
Heterogenous network showing an association between altered gut bacteria and metabolites in the WD group. (**A**) The figure shows the heterogeneous co-occurrence networks for the WD Control group. Circular nodes represent microbes in these networks, and squares represent metabolites. Microbe nodes (circles) have been colored by phylum (yellow = Firmicutes, brown = Actinobacteria, blue = Proteobacteria, violet = Bacteroidetes), with size proportional to their abundance. Metabolite nodes (squares) have been colored based on the sample set(s) where they are differentially abundant; otherwise, they are grey. Green edges represent positive correlations, and red edges represent negative correlations. The Fruchterman–Reingold algorithm has been used for visualization, keeping positively correlated entities in close proximity. Nodes have been labeled with their microbe or metabolite name, with a ranked centrality (importance) computed using Ablatio Triadum, which has been shown to uncover important driver, villain, and bridge nodes in signed and weighted biological networks. (**B**) Box plot showing network-specific metabolites that were altered. The box plots were constructed using log-transformed raw metabolite concentrations (based on ion counts). *p*-values were calculated by the Mann–Whitney test, where *p* < 0.05 was considered statistically significant. The black dots are used to denote the outlier data points.

In the WD group, we observed the presence of two KEGG pathways, both involving the metabolite cholate (taurobetamuricholate and tauroursodeoxycholate), and its connections with *Streptococcus thermophilus* and *Lactococcus lactis* (ranked #15) (Figure 6A). While pathway information was limited, it is noteworthy that *Lactococcus lactis*, along with cholesterol, has been associated with inflammatory gene expression [25]. In addition, there were 8 network-specific metabolites identified in the WD group(Figure 6B).

**Figure 7:**
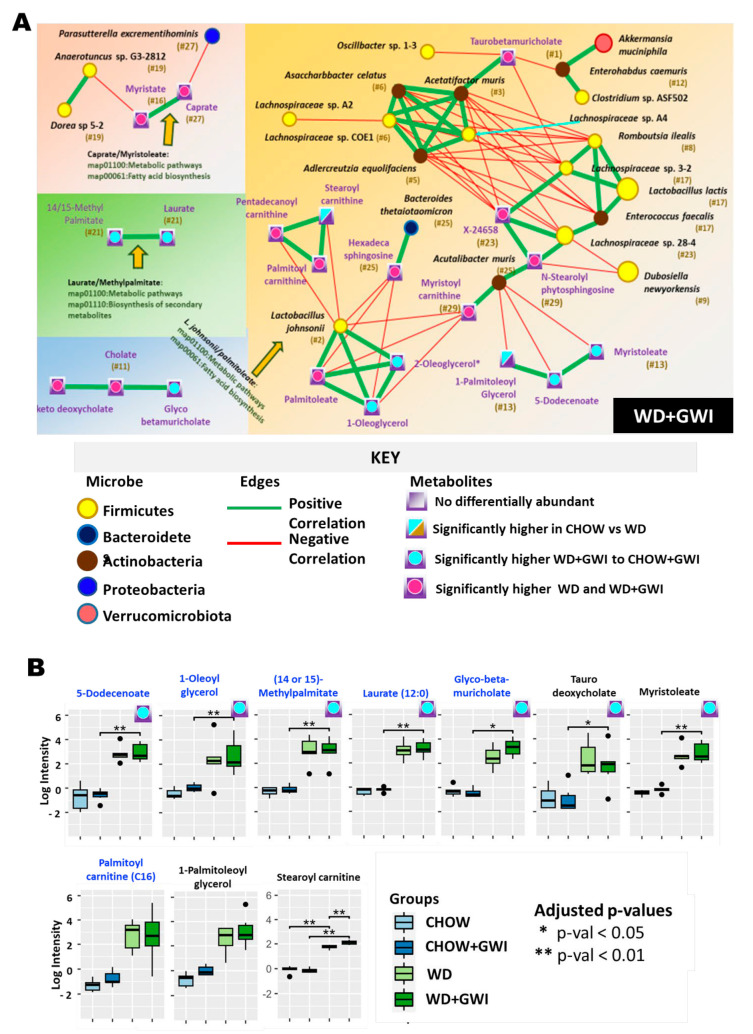
Heterogenous network showing an association between altered gut bacteria and metabolites in the WD + GWI group. (**A**) The figure shows the heterogeneous co-occurrence networks for the WD GWI group. Circular nodes represent microbes in these networks, and squares represent metabolites. Microbe nodes (circles) have been colored by phylum (yellow = Firmicutes, brown = Actinobacteria, blue = Proteobacteria, violet = Bacteroidetes), with size proportional to their abundance. Metabolite nodes (squares) have been colored based on the sample set(s) where they are differentially abundant; otherwise, they are grey. Green edges represent positive correlations, and red edges represent negative correlations. The Fruchterman–Reingold algorithm has been used for visualization, keeping positively correlated entities in close proximity. Nodes have been labeled with their microbe or metabolite name, with a ranked centrality (importance) computed using Ablatio Triadum, which has been shown to uncover important driver, villain, and bridge nodes in signed and weighted biological networks. Amber arrows point to any positive correlations that are also backed up by documented pathways in the database KEGG. (**B**) Box plot showing network-specific metabolites that were altered. The box plots were constructed using log-transformed raw metabolite concentrations (based on ion counts). *p*-values were calculated by the Mann–Whitney test, where *p* < 0.05 was considered statistically significant. The black dots are used to denote the outlier data points.

In the WD + GWI group, results showed three KEGG pathways, two of which are involved in fatty acid biosynthesis, which is interesting given that the Western diet is typically higher in fat (Figure 7A). One of these pathways is between two ranked metabolites, caprate (ranked #27) and myristate (ranked #16). The other pathway interestingly involved *Lactobacillus johnsonii*, which had initially appeared in the Chow + GWI network through its connection with palmitoleate [26]. It seems *Lactobacillus johnsonii* has a shift in its role in the WD + GWI group as compared to the Chow + GWI group. There were 10 network-specific metabolites identified in the WD + GWI group(Figure 7B).

## 3. Discussion

In our previous study, we showed that the administration of a Western diet that mimics the dietary pattern of present-day GW Veterans might exacerbate GWI symptoms via a potential gut–liver–brain axis. One of the consequential effects of gut dysbiosis is its wide-ranging effects on gut-derived metabolites. These metabolites often play a significant role in modulating inflammatory responses and the chronicity of symptoms [27]. In order to focus on a gut microbiome-directed approach in identifying modulators of inflammation and potential therapeutic targets [28], we performed a global metabolomics analysis using the fecal samples of the same groups, namely, Chow, Chow + GWI, WD, and WD + GWI. Metabolomics is a widely used omics approach that enables one to obtain an overview of the altered metabolites and study the major biochemical pathways that might be altered during certain disease development, and it can be used to identify novel therapeutic targets with broader implications [29].

In our present study, we found that the fecal metabolomic profile of the WD + GWI group was distinct from the Chow + GWI group (Figure 2B). The administration of representative GW chemicals PB and Per significantly altered the fold change of metabolites in the Chow + GWI group, which was further altered in the WD + GWI groups. We were also able to associate the altered gut bacteriome with metabolites and identify the biochemical pathways that were majorly altered in the Chow + GWI group compared to WD + GWI groups.

We found that *Lactococcus lactis,* which is a resident gut bacterium and maintains gut homeostasis by virtue of its anti-inflammatory actions [30], was positively associated with the cholate metabolite by heterogeneous network analysis (Figure 6A). Cholate and cholesterol metabolic pathways are activated due to diet-induced obesity by the activation of inflammatory genes, which results in hepatic inflammation [31]. This could be the reason for the increase in the abundance of this species in the WD groups. *Akkermansia muciniphila* had an indirect positive association with taurobetamuricholate, a bile acid, through the heterogeneous network analysis (Figure 7A). Taurobetamuricholate is a known agonist of the farnesoid X receptor found in the small intestine and liver and regulated by gut bacteriome, thus regulating the bile acid metabolism. Hence, alteration in this metabolite has serious consequences of causing liver inflammation, as was shown in a previous study [32], and the possible reason for increase in the abundance of *Akkermansia muciniphila* in WD + GWI group. 

The gut bacteriome is known to be influenced by varying factors such as age, gender, geographical location, diet, and activity, as well as environmental factors such as chemicals that include pesticides. Consequently, altered gut bacteria influence the metabolite levels of the host, which adversely affect the host’s health [8]. Pesticides such as pyrethroids (Per) and organophosphates (chlorpyrifos) are shown to have long-term effects on the gut bacterial population [33]. We have previously shown that GW-representative chemicals PB and Per significantly altered the gut bacterial population [5,7,9,28]. Metabolomic analysis of plasma from CD1 mice exposed to permethrin and pyridostigmine bromide after 150 days of exposure showed significant alteration in the lipid metabolism with the increase in sphingomyelin and phosphatidylcholine [14]. Our results were supported by these studies, as we observed similar alterations of the lipid metabolism in different forms of lipid metabolites, but the changes persisted even after 20 weeks of exposure.

Permethrin exposure is known to alter the relative abundances of gut bacterial species responsible for polyamine metabolism and short-chain fatty acid production [33]. Co-exposure to PB and Per is reported to decrease the activity of lipase, which led to an increase in total lipids [14]. In our global metabolomic analysis, we found several metabolites that were significantly changed. Adenosine, which is essential for the construction of RNA and also serves as a potent drug to treat heart disease, was found to be significantly decreased in all the groups compared to the Chow group [17]. Oleoyl ethanolamide, palmitoyl ethanolamide, and margaroyl ethanolamide are associated with endocannabinoid metabolism [15,16]. All these acylethanolamides were also found to be significantly decreased in the Chow + GWI, which explains the increase in the expression of neuroinflammatory and neurodegenerative markers in this group [9]. These acylethanolamides help in protecting the host brain health, decrease neurodegeneration, and also imparts anti-inflammatory effects [34]. Although the concentration of these metabolites was significantly lower in the Chow + GWI group, the concentration was markedly decreased in the Western diet cohort, suggesting that diet had a significant impact on these metabolites. Taurocholate is known to trigger adaptive cytoprotection in the gut [35] and is the main product of cholesterol catabolism [36], a key component of the Western diet [37]. The Chow + GWI group showed an increased concentration of taurocholate compared to the Chow group; moreover, it was increased in both the WD and WD + GWI groups though the changes were not significant. Another metabolite, 1-docosahexaenoylglycerol, had elevated levels in the Chow + GWI and WD + GWI groups (research is limited on this secondary metabolite). Concentrations of the following metabolites that were potentially beneficial or toxic to the host health were observed to be increased in the WD group compared to the WD + GWI group. The metabolite 3-ureidopropionate, which is involved in pyrimidine metabolism, was reported to be a potential neurotoxin [38]. An increase in propionylcarnitine has been equated with vitamin B12 deficiency [39]. Potential beneficial metabolites were also observed to be increased in the WD group. The metabolite 3-(4-hydroxyphenyl) lactate belongs to tryptophan and tyrosine metabolic pathways, which are natural antioxidants [40]. N-acetyl aspartate has been used as a biomarker for a healthy human brain and has been shown to be reduced following brain injury [41]. In the WD + GWI group, increased concentrations of the following metabolites, compared to the Chow + GWI group, and having pathological implications were observed. L-urobilin is the oxidized urobilinogen and byproduct of bilirubin degradation [42]. Elevated levels of urobilinogen are currently used as a test for both liver disease and hemolytic anemia [43], the latter of which has symptoms (fatigue, headaches, dizziness) that overlap with those of GWI [44]. Increased red blood cell dysfunction has already been reported in patients with GWI [45]. Oleoylcarnitine has been shown to be an inhibitor of adenine nucleotide translocase activity in arterial cells [46]. p-cresol sulfate, a potent neurotoxin, could regulate synaptic plasticity via brain-derived neurotrophic factor at low levels. However, elevated levels of p-cresol result in neuroinflammation and oxidative stress in the brain and are evident in GWI pathophysiology [47]. The metabolites observed to be increased in the CHOW + GWI and WD + GWI could be further studied for their potential as a biomarker for GWI chronicity.

We advanced our previously reported studies by introducing a novel approach. The co-occurrence heterogeneous network analyses for all the experimental groups were performed to study the association between the altered gut bacterial species and the altered metabolites. Results from this analysis showed that certain bacterial species were associated with essential host metabolites. There were certain cohort-specific metabolites obtained that could aid in determining the potential biomarkers or therapeutic targets, thus significantly advancing the present knowledge about the GWI pathophysiology. *Parasutterella excrementihominis* and *Akkermansia muciniphila* dissociated from the main network in the Chow + GWI group. *Bacteroides thetaiotaomicron* formed a new network in that group. N-carbamoyl aspartate, which is involved in the synthesis of glutamate, an important neurotransmitter and associated with gut commensal *Adlercreutzia equolifaciens,* could be used as a novel biomarker for GWI conditions. Neuronal and cognitive dysfunctions are important symptoms in GWI; hence, further studies involving N-carbamoyl aspartate would be beneficial in understanding the neuronal dysfunctions due to GW chemical exposures. Studying the bacteria-metabolite networks of WD and WD + GWI groups, we observed that *Akkermansia muciniphila* was only present in the WD + GWI network. *Clostridium cocleatum, Staphylococcus xylosus, Streptococcus thermophilus,* and *Ruthenibacterium lactatiformans* were only observed in the WD group network. The rank of *Parasutterella excrementihominis* decreased from #1 in the WD group to #27 in the WD + GWI group, indicating the influence of both diet and GW chemicals. Stearoyl carnitine was significantly increased in the WD + GWI group. This metabolite is involved in fatty acid metabolism and chronic fatigue syndrome [48]. Stearoyl carnitine could be used as a potential biomarker in GWI due to the similarity in its symptoms with chronic fatigue syndrome [28].

**Limitations of the study:** Although we obtained novel and interesting results in terms of bacteriome–metabolome associations, there remained certain limitations that need to be addressed. We had only six mice per experimental group; hence, an increase in the sample size would aid in producing a more comprehensive network analysis for the cohorts. The network analysis is an association study; hence, there is a need to measure the serum level of the metabolites to confirm the changes in the metabolite. The inclusion of animal behavior, anxiety, and memory tests of the experimental mice in the GWI model would increase the translatability of the results to the present-day GW Veterans. We would be including the behavioral tests along with proposed metabolomic analyses in our future studies. There is also a need to analyze the metabolomic profiles of the GWI Veterans to corroborate the data obtained in this preclinical study. An in-depth understanding of the influence of gut bacteriome on the host metabolomic profile could be further confirmed by conducting the same experiments using germ-free mice models or in mice with antibiotics-induced gut bacteriome depletion. The GWI murine models presently used to study GWI pathophysiology cannot be deemed perfect. There is also a need to incorporate other environmental chemicals such as organophosphates along with the present combination of representative GW chemicals, as the GWI condition is a result of complex combinatorial environmental toxin exposure. A study of the routes of administration of the GW chemicals in order to closely mimic the condition to that of the GW Veterans remains a viable future option in this field to advance our understanding of the gut microbiome–metabolome interaction. Finally, we would like to state that this is a proof-of-concept work that needs to be evaluated in a GW Veteran cohort. We are in the process of conducting this research, but the proper materialization of the concepts will take time.

**Conclusion:** In conclusion, this study holds significant value, since it has not only shown the gut bacterial–metabolite association but also provided information about certain novel metabolites, which could be used to study their ability in ameliorating GWI symptom persistence. It also provides important clues about altered biochemical pathways, which could be further studied to understand the GWI pathophysiology and, most importantly, its chronicity. The results from this study could be used in general to understand pyrethroid and organophosphate toxicity when used indiscriminately. In order for the results to be translatable in GWI Veterans, we need to perform a global metabolomics analysis in a GW Veteran cohort. The overlap in the pathology of chronic fatigue syndrome suffered by the aging population and GWI symptoms further widens the translatability and scope of these results in people suffering from chronic fatigue syndrome as well.

## 4. Materials and Methods

### 4.1. Animals

Pathogen-free, wild-type, male, adult (10 weeks old) C57BL/6J mice were purchased from Jackson Laboratories (Bar Harbor, ME, USA). All mice were housed in a temperature-controlled (22–24 °C) room with a 12 h light/12 h dark cycle after arrival and had ad libitum access to both food and water. All mice experiments mentioned in this present study were approved by the University of South Carolina (Columbia, SC, USA) and conducted by strictly following the guidelines implemented by the National Institutes of Health (NIH) for humane care and use of laboratory animals and local Institutional Animal Care and Use Committee (IACUC) standards (protocol no. 2419-101345-072318 approved on 7/23/2020).

### 4.2. Mouse Model of Gulf War Illness

Upon arrival, all mice were first acclimatized for a week and then randomly distributed into four experimental groups with 6 mice per group (n = 6/group). Both the first (denoted as Chow) and third (denoted as WD) groups of mice were dosed with vehicle (0.6% dimethyl sulfoxide (DMSO)) for only two weeks, whereas the second (denoted as Chow + GWI) and fourth (denoted as WD + GWI) groups were administered with a mixture of GW chemicals PB (2 mg/kg body weight; diluted in phosphate-buffered saline) and Per (200 mg/kg body weight; diluted in DMSO and phosphate-buffered saline) tri-weekly for two weeks via an oral gavage route. During the initial two weeks of the vehicle or GW chemical administration, all mice groups were fed only the chow diet (Teklad, Madison, WI, USA). After that, only the Chow and Chow + GWI groups were continuously fed with the chow diet, whereas both WD and WD + GWI groups were fed with the Western diet (Research Diets, New Brunswick, NJ, USA) for a continuous 20-week period. The Western diet (Research Diets, Cat#12079B) used for this study contained 17% kcal protein, 40% kcal fat, and 43% kcal carbohydrate in its composition. All mice were euthanized at the end of the study, and fecal pellets were collected for bacteriome and metabolome analysis from each experimental mouse.

### 4.3. Bacteriome Analysis

Bacteriome analysis was performed by the vendor CosmosID Inc. (Germantown, MD, USA). In brief, total DNA samples from mouse fecal pellets were isolated and purified using the ZymoBIOMICS (Irvine, CA, USA) Miniprep kit. Then, total DNA was quantified using the Qubit dsDNA HS assay (Thermofisher, Waltham, MA, USA). After that, DNA libraries were prepared using the Illumina (San Diego, CA, USA) Nextera XT library preparation kit. Illumina HiSeq 4000 and Illumina NextSeq 550 platforms were used to perform whole-genome sequencing for all mice samples. As optimized by the vendor, 2 × 150 bp of read length and an average insert size of 1400 bp were used for the sequencing process. The preparation of DNA libraries was performed using the Nextera XT DNA Library preparation kit (Illumina) with Nextera index kit (Illumina) with a total DNA input of 1 ng. Following that, the fragmentation of genomic DNA was performed using a proportional amount of Illumina Nextera XT fragmentation enzyme. Combinatory dual indexes were added to each sample, followed by 12 cycles of PCR to construct libraries. The purification of DNA libraries was performed using AMpure magnetic beads (Beckman Coulter, Brea, CA, USA) and eluted in QIAGEN EB buffer. Quantification of DNA libraries was performed using a Qubit 4 fluorometer and QubitTM dsDNA HS assay kit. Upon data arrival, raw data were backed up to Amazon AWS and run through fastqc, and a multiqc report was generated. The multiqc report was checked to ensure read depth thresholds were met, and that there were no abnormalities with read quality, duplication rates, or adapter content. Taxonomic results were checked on the vendor’s COSMOSID-Hub Microbiome platform to ensure there were no contamination or barcoding issues.

### 4.4. Metabolomics

The metabolomics profile was generated by Metabolon (Metabolon Inc., Morrisville, NC, USA) using their global metabolomics platform with fecal pellets collected from all the experimental mice. In brief, samples were prepared first using the automated MicroLab STAR^®^ system (Hamilton Company, Reno, NV, USA). Then, proteins and small molecules were removed by precipitation with methanol, followed by centrifugation to ensure the recovery of various metabolites present in the samples. All the collected extracts were then subjected to an Ultrahigh Performance Liquid Chromatography–Tandem Mass Spectroscopy (UPLC-MS/MS) method. All methods utilized a Waters ACQUITY ultra-performance liquid chromatography (UPLC) and a Thermo Scientific Q-Exactive high-resolution/accurate mass spectrometer interfaced with a heated electrospray ionization (HESI-II) source and Orbitrap mass analyzer operated at 35,000 mass resolution. The sample extract was dried and then reconstituted in solvents that contained a series of standards at fixed concentrations to ensure injection and chromatographic consistency. Raw data were extracted, peak-identified, and QC processed using Metabolon’s hardware and software. Metabolites were identified by comparing with the library based on authenticated standards that contain the retention time/index (RI), the mass-to-charge ratio (m/z), and chromatographic data (including MS/MS spectral data) on all molecules present in the library. Finally, the quality control and curation processes were performed to ensure accurate and consistent identification of true chemical entities and to remove those representing system artifacts, misassignments, and background noise.

### 4.5. Metabolomics Data Analysis

The box-and-whisker plots were constructed using log-transformed raw metabolite concentrations (based on ion counts). Our network analysis began from two separate datasets: (1) a set of relative microbial abundances in each sample; and (2) a set of metabolite concentrations mapped to a normal distribution around a zero mean. With microbial abundances tending to be sparse with a smaller range of values (mostly zero or near-zero), we used SparCC [49] (*p* = 0.05) to compute microbe–microbe correlations, which has been proven to reduce compositional effects in sparse datasets. Metabolite concentrations tend to be complete, and their normalized values will have a range larger than [0, 1], so we used Spearman (*p* = 0.05) correlations for metabolite–metabolite correlations, which used ranks to reduce dependence upon magnitude. For heterogeneous (microbe–microbe) correlations, we first computed ranks of microbes and metabolites separately and then computed Spearman correlations (*p* = 0.05) using both sets of ranks.

These results were visualized as a correlation network. Correlation networks measured the co-occurrence, or the tendency of two entities to appear together or separately in samples. Microbial co-occurrence networks (MCN) [50] can estimate ecological relationships (i.e., cooperation, competition) within a microbial ecosystem [51]. Incorporating metabolites into MCNs to form a heterogeneous network delves into the mechanisms behind these relationships, increasing the depth of the analysis and potentially leading to valuable conclusions regarding microbes producing and consuming nutrients and/or toxins.

### 4.6. Statistical Analyses

Statistical analyses for all the plots were performed using the Mann–Whitney test. *p* < 0.05 was considered to be statistically significant and marked with one star in the figures. For data sets that had *p* < 0.01, we have denoted them with two stars.

## Data Availability

The data presented in this study are available on request from the corresponding author.
